# *Leporinus elongatus* (Characiformes, Anostomidae): complete mtDNA sequence of an economically important fish from the Paraná and La Plata river basins

**DOI:** 10.1080/23802359.2017.1318685

**Published:** 2017-05-09

**Authors:** Alessandra Gomes Bedore, Leonardo Cardoso Resende, Anderson Oliveira do Carmo, Daniela Nuñez Rodriguez, Ana Paula Vimieiro Martins, Evanguedes Kalapothakis

**Affiliations:** Departamento de Biologia Geral, Instituto de Ciências Biológicas – Universidade Federal de Minas Gerais, Avenida Presidente Antônio Carlos, Belo Horizonte, Brasil

**Keywords:** Mitochondrial genome, ‘piapara’, ‘piau verdadeiro’, next-generation sequencing

## Abstract

*Leporinus elongatus* is an important commercial fish found in the La Plata and Paraná River basins. Next-generation sequencing was used to sequence the mitochondrial DNA (mtDNA) of *L*. *elongatus.* The mtDNA was assembled using the CLC Workbench software v. 9.0 and subsequently aligned to other 10 complete fish mitochondrial sequences to enable phylogenetic analysis using MEGA 7.0. The complete mtDNA molecule had 16,784 bp and its GC content was 43%. The mtDNA structure was similar to that of other vertebrates: two ribosomal RNA, 22 transfer RNA, 13 protein-coding genes, and a D-loop region containing 1115 bp. Phylogenetic analysis yielded a tree with high bootstrap value that was coherent with the current phylogeny proposed for Characiformes.

*Leporinus elongatus* is one of 110 species of Anostomidae family, order Characiformes, being highly abundant and having a fishery importance in Paraná and La Plata river basins (Vaz et al. 2000). Commonly known as ‘piapara’, it is considered as an omnivorous species, feeding on plants, insects and molluscs (Langeani & Rêgo 2014).

For fish species considered as highly migratory, like ‘piapara’, populations can be concentrated on areas such as stocks, natural gene banks or management units (Moritz 1994; Toledo-Filho et al. 1992), making their migration process negatively impacted by dam constructions (Agostinho et al. 2007). Stockpiling or re-stocking is one of the main management actions applied worldwide to restore migratory fish stocks.

With the aim of improving these management measures, emerged mitochondrial and nuclear molecular markers, enabling analysis of population structure and genetic diversity, minimizing possible inbreeding effects.

A sample of muscle was extracted of one specimen obtained in EAVG (CEMIG) Minas Gerais State, Brazil (20°09′09″S, 48°01′56″W) and stored at the Tissue and DNA Collection facility of the Universidade Federal de Minas Gerais (deposit code: UFMG-BDT-PP000005). The genomic library was constructed using Nextera DNA Library Preparation kit (Illumina Inc., San Diego, CA) and sequenced with MiSeq sequencer (Illumina) with a paired-end 300 bp strategy. The CLC Workbench software v. 9.0 (CLC Bio-Qiagen, Aarhus, Denmark) was used for *de novo* assembly, and the mitochondrial genome was annotated using the MitoFish webserver (Iwasaki et al. 2013). Complete mtDNA sequences from ten other species were retrieved from GenBank. Sequences were subsequently aligned to enable phylogenetic analysis with MEGA version 7.0.14 (Kumar et al. 2016), using a maximum likelihood method with 1000 bootstrap replications and the Tamura-Nei model (Tamura & Nei 1993) for nucleotide substitution (Figure 1).

**Figure 1.**
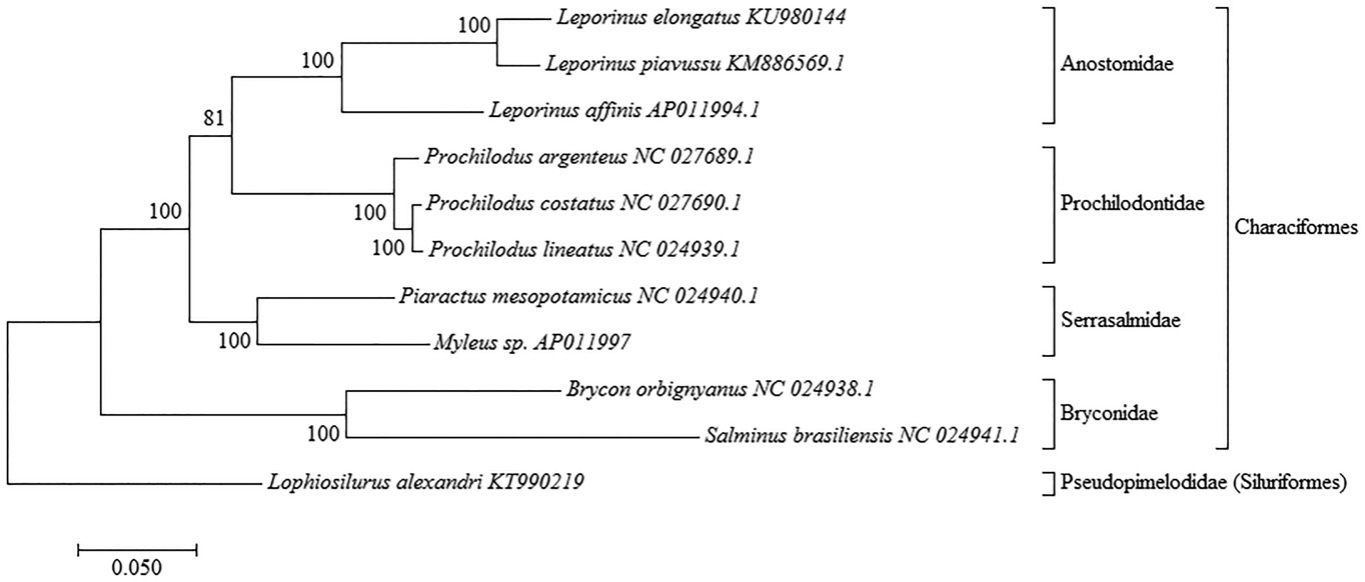
Molecular phylogenetic analysis inferred using the maximum likelihood method assessed by Tamura–Nei model (Tamura & Nei 1993) with 1000 bootstrap replications. The D-loop region was excluded from this analysis because it is considered to be highly variable (Gonder et al. 2007). Phylogenetic analyses were conducted for *Leporinus elongatus* (GenBank accession no. KU980144) and complete mtDNA of 10 others species: *Leporinus piavussu* (KM886569), *Leporinus affinis* (AP011994), *Prochilodus argenteus* (KR014816), *Prochilodus costatus* (KR014817), *Prochilodus lineatus* (KM245045), *Myleus* sp. (AP011997), *Piaractus mesopotamicus* (KM245046), *Salminus brasiliensis* (NC024941.1), *Brycon orbignyanus* (NC024938.1) and *Lophiosilurus alexandri* (KT990219). The phylogenetic tree with the highest log likelihood is shown, and bootstrap value in which associated taxa clustered together is described next to the branches. The tree was rooted on *Lophiosilurus alexandri* KT990219 (Order Siluriformes, Family Pseudopimelodidae). Genus *Leporinus* was confidently recovered as a monophyletic group as well as the Family Anostomidae with a high bootstrap value (BSP =100%). Families Anostomidae and Prochilodontidae (Superfamily Anostomoidea) were also recovered as a monophyletic group, but with lower BSP (81%) (Chagas et al. 2016).

The mitochondrial genome of *L. elongatus* was 16,784 bp (GenBank KU980144) solved with 205.16 folds of coverage. The GC content of the total sequence was 43%, with individual base content being: 29.9% A, 15.7% G, 27.1% T and 27.3% C. The mtDNA genome contains 2 rRNA and 22 tRNA genes, 13 protein-coding genes (PCGs) and a control region (D-loop) with 1115 bp, being consistent with mitochondrial genome structure of the fishes (Brandão-Dias et al. 2016; Carmo et al. 2016; Chagas et al. 2016; Núñez-Rodriguez et al. 2016; Pimentel et al. 2016; Resende et al. 2016; Siqueira et al. 2014). Only gene *COI* started with GTG codon, differing from all other PCGs that display the usual start codon ATG. Twelve of the PCGs were located on the heavy strand, and one PCG (ND6) was located on the light strand. Only five out of 13 PCGs (*ND1*, *ND2*, *ATPase8*, *ND4L* and *ND5*) contained a TAA stop codon; four (*COII*, *ND3*, *ND4* and *Cytb*) displayed incomplete termination codon (T–); three genes (*ATPase6*, *COIII* and *ND6*) displayed the incomplete TA- stop codon. Only *COI* presented AGG as stop codon, similar to others Characiformes (Siqueira et al. 2014; Brandão-Dias et al. 2016; Pimentel et al. 2016); considered a vertebrate evolution (Osawa et al. 1989).
